# Diabetic Ketoacidosis at Onset of Pediatric Type-1 Diabetes Triggered by Covid-19: An Original Case Report

**DOI:** 10.7759/cureus.13958

**Published:** 2021-03-17

**Authors:** Salma Benyakhlef, Wahiba Abdellaoui, Abir Tahri, Siham Rouf, Hanane Latrech

**Affiliations:** 1 Department of Diabetology and Endocrinology, Mohammed VI University Hospital, Mohamed I University, Oujda, MAR; 2 Department of Epidemiology, Clinical Research and Public Health, Mohamed I University, Oujda, MAR

**Keywords:** covid-19, pediatric t1dm, diabetic ketoacidosis

## Abstract

During the course of this year, scientific research has revolved around coronavirus disease 2019 (COVID-19), with much uncertainty surrounding its pathogenesis, complications, and mortality as well as limited pediatric evidence. The exact relationship between COVID-19 and new-onset type 1 diabetes, especially in children, remains an unresolved issue. Our case exhibited a unique presentation of diabetic ketoacidosis (DKA) triggering COVID-19 diagnosis at this age. A three-year-old Moroccan boy was admitted with a history of acute dyspnea accompanied by vomiting and weight loss, leading to a diagnosis of DKA. However, evident respiratory distress signs did not disappear, even though glycemic levels were normalized and acidosis resolved. The boy subsequently tested positive for SARS-CoV-2 (severe acute respiratory syndrome coronavirus 2), with specific biological and radiological findings. Our clinical case potentially augmented the global registry of COVID-19-related diabetes (CoviDiab project) cases and provides a basis for further studies that seek to elucidate the correlation between new-onset type 1 diabetes and SARS-CoV-2 in the pediatric population.

## Introduction

More than a year has passed since the first cases of coronavirus disease 2019 (COVID-19) were reported in Wuhan, China, killing more than one million people worldwide, especially those with serious comorbidities such as diabetes [1]. Morocco is currently experiencing a second wave of COVID-19 infections, as recently observed in other countries, and evidence regarding the onset of type 1 diabetes mellitus (T1DM) in children in light of this pandemic is limited [1]. However, epidemiological reviews have steadily found a decreased risk of evolving severe features in children in comparison with adults, despite the increase in diabetic ketoacidosis (DKA) incidence during childhood, particularly severe forms. This may be explained primarily by delayed diagnosis, inter alia, because of a lack of caregivers-seeking behavior [1]. Reporting cases where there are neither valid cohorts nor a complete SARS-CoV-2 infection background may help in establishing the basics of hyperglycemic mechanisms that correlate with COVID-19 and considering therapeutic and prevention pathways. To date, defined guidelines on managing pediatric cases of COVID-19 have neither been conceptualized nor availed to healthcare workers [1]. In this paper, we considered an original case of a three-year-old Moroccan boy who presented with diabetic ketoacidosis as the initial feature of his diabetes mellitus that was triggered by SARS-CoV-2, which was diagnosed following the non-resolution of respiratory distress, despite resolution of the acute episode.

## Case presentation

A three-year-old boy was referred to the emergency department of Mohammed VI University Hospital in Oujda due to the development of acute dyspnea accompanied with asthenia and vomiting, without abdominal discomfort.

This boy was the youngest of three siblings, born at full term to a non-consanguineous parents, without any infectious background, breastfed for 13 months, and vaccinated in conformance to the Moroccan immunization schedule. His grandparents on both sides had type 2 diabetes mellitus; however, no documented genetic disorder was noted. The parents faithfully narrated a two-week history of polyuria, polydipsia, and asthenia, developments that kept him away from kindergarten, with a loss of approximately 2 kg during the previous month, inconsistent with his polyphagia. His vital parameters were monitored: temperature was 38°C and respiratory rate up to 50 breaths/min without Kussmaul breathing; however, he had evident signs of respiratory distress, particularly nasal flaring and chest retractions. Initially, his oxygen saturation was approximately 93% under 3L of oxygen provided by a simple facial mask. His heart rate was 99 beats/min without any signs of dehydration. His throat was swollen, and he had developed conjunctival hyperemia. The child’s capillary blood glucose was assessed, reaching 16,67 mmol/L, and his urine ketone and glucose levels each were 3+. His pulmonary auscultation was clear, and his neurological examination was normal. The remainder of his physical examination was uneventful.

**Figure 1:**
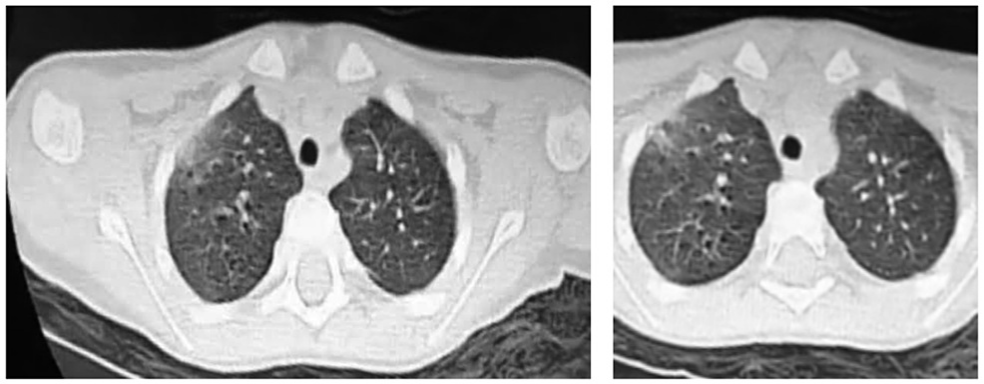
Axial images of the present case’s chest CT showing ground-glass opacities with consolidations

The diagnosis of mild DKA was obviously assessed (pH: 7.25, bicarbonate: 13.2 meq/L, and decreased alkaline reserve: 8 mmol/l). His serum electrolytes were analyzed, and hypokalemia was detected (K: 2.7 mmol/L). Additionally, his C-reactive protein (CRP) level was negative (1.15 mg/L), while his procalcitonin level increased to 3,39 ng/L. His complete blood count revealed lymphocytopenia (600), and fibrinogen (2.2) [2-4], platelet count, and D-dimer level were within their normal ranges. In the intensive care unit, the patient immediately received isotonic fluids and continuous insulin infusion at 0.1 u/kg/h, with hypokalemia correction in addition to antipyretics. Despite the DKA resolution, tachypnea (respiratory rate: 40 breaths/min) and signs of respiratory distress were observed, requiring non-invasive ventilation. Considering the COVID-19 pandemic, a reverse-transcription polymerase-chain-reaction (RT-PCR) nasopharyngeal swab was performed and tested positive for SARS-CoV-2, in a manner similar to his parents, while they were asymptomatic. Thereafter, the patient underwent a chest computed tomography scan, revealing bilateral ground-glass opacities, especially in the subpleural region with left consolidations, and graded COVID-19 Reporting and Data System 4 (CO-RADS 4) by the radiologist (Figure 1). After 48 hours in intensive care, his clinical symptoms improved gradually, and he started breathing room air and was moved to an isolation COVID-19-care unit with his mother. The basal bolus insulin regimen was initiated, and diabetes education was provided and evaluated through follow-up sessions, even after hospital discharge. The boy’s HbA1c value was 10.3 %, and his islet autoantibodies were positive. After 10 days in the hospital, the patient and his mother were discharged with quarantine recommendation.

## Discussion

COVID-19 pandemic affects individuals of all ages. Conventionally, it is usually observed in adults, unlike in children and adolescents, who can display misleading symptoms, such as gastrointestinal complaints. Thus, diagnosis may be delayed, whether of the viral infection or of T1DM itself, which is responsible for increasing the DKA condition [2].

Patients with type 2 diabetes mellitus (T2DM) infected with SARS-CoV-2 have been widely reported and have been at great risk since SARS-CoV-2 outbreak, mostly because of an array of comorbidities and poor glycemic control. However, data regarding T1DM in the pediatric population are scarce, and the relationship between SARS-CoV-2 and T1DM remains unclear. In fact, only some cases have been outlined, despite recent enthusiasm regarding bidirectional studies [3]. To the best of our knowledge, only four pediatric cases presenting with new-onset DKA induced by SARS-CoV-2 have been reported according to our literature review (Table 1).

**Table 1:** Published cases of COVID-19 revealed by DKA at T1DM onset DKA: diabetic ketoacidosis; T1DM: type 1 diabetes mellitus

Patient	Clinical Presentation	Laboratory Testing	Imaging results	Treatment	Follow-up
Present case	Male, three years old, polyuria, polydipsia, dyspnea *No Kussmaul respiration	Glucose: 3 g/dL; pH: 7.25; bicarbonate: 13.2 meq/L; HbA1c: 10.3%; K: 2.7 meq/L; CRP: 1.15 g/L; PCT: 3.39 ng/L; fibrinogen: 2.2 g/L; D- dimer: 3 ng/mL; anti-GAD ab: 60 IU/mL (<5); SARS-CoV-2; RT-PCR: positive	Chest computed tomography: bilateral ground-glass opacities especially in subpleural region + left consolidations	Insulin-infusion protocol switched to subcutaneous insulin regimen, non-invasive ventilation, potassium chloride	Basal-bolus insulin regimen
Rabizadeh et al. [2]	Male, 16 years old, polyuria, polydipsia, abdominal pain, weight loss T: 38.2 °C, oxygen saturation: 94% on room air, no Kussmaul respiration	Glucose: 5.12 g/dL; pH: 6.9; bicarbonate: 8 meq/L; C-peptide: 0.25 ng/mL (0.78–1.89); HbA1c: 8.5%; CRP: 44 g/L; SARS-CoV-2 RT-PCR: positive	Chest computed tomography: normal	Insulin-infusion protocol switched to subcutaneous insulin regimen; potassium chloride; hydroxychloroquine+Lopinavir and ritonavir association)	Basal-bolus insulin regimen
Soliman et al. [4]	Male, eight months old, lethargy, fever, vomiting, tachypnea, 10% dehydration, no Kussmaul respiration	Glucose: 5.71 g/dL; pH: 7.08; bicarbonate: 7 meq/L; C-peptide: 0.43 ng/mL (0.5–5.5); HbA1c: 12.9%; K: 5.6 meq/L; CRP: 4.2 g/L; anti-GAD ab: 34.9 IU/mL (<5); SARS-CoV-2 RT-PCR: positive	Chest radiograph: normal	Intravenous insulin therapy	Basal-bolus insulin regimen
Naguib et al. [5]	Female, eight years old, polyuria, polydipsia, anorexia, weight loss, fever, vomiting, rash, conjunctivitis, cough + rhinorrhea, no Kussmaul respiration, multisystem inflammatory syndrome	Glucose: 4.29 g/dL; pH: 7.3; bicarbonate: 14 meq/L; HbA1c: 12%; K: 3.4 meq/L; CRP: 2.41 g/L; fibrinogen: 8.16 g/L; D-dimer: 955 ng/mL; SARS-CoV-2 RT-PCR: not detected; SARS-CoV-2 IgG: 9.7 (N: <0.7)	Chest X-ray: low lung volumes, trace pleural effusions	Subcutaneous insulin infusion: total dose: 1.4 UI/kg/day, methylprednisolone 1 mg/kg BID for five days, IVIG 2g/kg, infliximab 10 mg/kg	After one month: HbA1c: 7.8%; Total dose of insulin: 1.1 UI/kg/day
Daniel et al. [6]	Female, 15 years old, lethargy, abdominal pain, vomiting, tachypnea (RR: 40/min), Kussmaul respiration	Glucose: 4.14 g/dL; pH: 6.9; bicarbonate: 2 meq/L; HbA1c: 13.5%; SARS-CoV-2 RT-PCR: positive	Chest radiograph: low lung volume + mild bilateral haziness	Non-invasive ventilation, insulin-infusion protocol, hydroxychloroquine	Basal-bolus insulin regimen

In light of this, many reports have revealed an increase in severe presentations of new-onset T1DM in children during this pandemic era. Additionally, a review of the Spanish SARS-CoV-2 epicentre included cases of younger individuals [7] as compared to prior years, and, accordingly, our case involved a three-year-old boy. In fact, Italy recorded 44.3% of severe cases of DKA, compared to 36% during the same period in 2019 [8]. UK listed 70% of DKA cases, and approximately 52% were severe [9]. 

Comprehending the reason for DKA in our case is more laborious than it seems. Actually, infection may also be considered in DKA, and viral exposure along with genetic background are defined in autoimmune T1DM pathogenesis; however, this viral contact should normally disappear within months or years [9]. Moreover, the description of the correlation between SARS-CoV-2 and renin-angiotensin-aldosterone system (RAAS), which exhibits the role played by angiotensin-converting enzyme 2 (ACE2) receptor as the binding piece of SARS-CoV-2 definitely incriminates this virus in β-pancreatic cell dysfunction accelerating the DKA process [9]. Nonetheless, to date, the intensity of ACE2 (angiotensin-converting enzyme 2) expression in β-cells is still contested, and the possibility of direct β-cell aggression cannot be proven in the absence of effective pancreatic examination of the infected patients [3]. Nevertheless, decreased ACE2 expression tends to reduce angiotensin II degradation, while aldosterone and renal potassium loss increase. This mechanism contributes to hypokalemia and possibly requires further potassium supplementation. Moderate hypokalemia was detected in our case and corrected using intravenous potassium infusion [9]. Furthermore, the relationship between SARS-CoV-2 and RAAS is involved in DKA treatment through the increase in respiratory distress in the case of disproportionate fluid resuscitation, because of the participation of angiotensin II in worsening pulmonary vascular permeability [4].

Despite fact that pathognomonic laboratory features have not yet been clearly detailed among pediatric populations with SARS-CoV-2, Xia et al. defended the fact that the elevation of procalcitonin must be considered as it is uncommon in adult patients [10]. Apparently, in the same report, the most frequent laboratory findings were as follows: discordant WBC counts, normal or momentarily elevated CRP, and increased platelet count (PCT). Our patient had increased PCT (3.39 ng/L), normal CRP level, and lymphopenia.

Overall, radiological examinations are also helpful in the diagnosis pathway, and chest CT aspects resemble those of adults. Nevertheless, Xia et al. pointed out consolidations with surrounding halo signs as a classic sign in infected children [10]. Our patient exhibited bilateral ground-glass opacities, especially in the subpleural region with left consolidations, graded CO-RADS 4.

## Conclusions

COVID-19 is a worldwide health emergency, with many uncertainties in terms of its pathogenesis, complications, mortality rate, and adequate treatment. To diabetologists, the correlation between COVID-19 and new-onset diabetes, especially in children, remains unresolved. The case described herein exhibits a unique presentation of DKA triggering COVID-19 diagnosis at this age. Therefore, our clinical case potentially augments the global registry of cases with COVID-19-related diabetes (CoviDiab project) and provides a basis for further research that seeks to understand the correlation between diabetes and COVID-19 in the pediatric population. Currently, more attention is required to enhance COVID-19-linked DKA prognosis, and sound diabetes education should be provided to the affected child and his/her family.
